# Preparation and Property of Perfluoropolyether Emulsions

**DOI:** 10.3390/polym11060932

**Published:** 2019-05-29

**Authors:** Dianlong Zhang, Yiqiang Zhang, Yanyun Bai, Xiumei Tai, Wanxu Wang, Guoyong Wang

**Affiliations:** 1 Department of Chemistry, Shanxi Datong University, Datong 037009, China; Zhandl@yeah.net; 2 China Research Institute of Daily Chemical Industry, 34 Wenyuan Street, Taiyuan 030001, China; 18035660723@163.com (Y.Z.); 13503517996@163.com (Y.B.); 18353111955@163.com (X.T.); wang3063025@126.com (W.W.)

**Keywords:** PFPE, droplet size, Ostwald ripening, application performance

## Abstract

Perfluoropolyether (PFPE) glycerol emulsions were prepared. Three different green surfactants (AES (sodium laureth sulfate), APG (alkyl polyglycoside), and SDS (sodium dodecyl sulfate)) were chosen to emulsify the PFPE. Their properties and performance in shampoo were also investigated. Centrifuge stability measurements show that three PFPE emulsions have good stability. They are stable for 60 min when the centrifugal speed is 6000 r/min. In addition, a change of droplet size was observed with time. Moreover, its rheological properties and application performance was studied. The AES emulsion was the most stable emulsion and it was found to improve the slip and lubricity performance of the cotton, so it has potential applications in shampoo.

## 1. Introduction

Silicone oil, polymerized siloxane with organic side chains, is one of the most important polymer materials. Silicone oil has attracted much scientific and commercial interest because of its relatively high thermal stability and lubricating properties [1]. As of now, silicone oil is widely used in detergents as a conditioning agent. This is because silicone oil can smooth out scales and reduce the friction of the hair, so that the hair is smooth and easy to comb [1]. However, according to research [2], amino silicone oil shows obvious ecological acute toxicity. At the same time, amino silicone oil is mainly discharged into the environment through the sludge treatment of sewage. Only a small amount of amino silicone oil is discharged into water in the environment, and sediments, along with the waste water and the amino silicone oil, cannot be effectively biodegraded during the anaerobic biochemical treatment of wastewater. Moreover, since silicone oil is insoluble in water, excessive deposition on the scalp may cause hair follicles to clog, affect the health of the scalp and hair, and may even lead to scalp inflammation and hair loss [1]. Thus, developing a new type of material to replace silicone oil will become increasingly important.

Perfluoropolyether (PFPE) is another kind of special liquid material. It is colorless, tasteless, no-poisonous, hypo-allergenic, and has excellent handling properties, excellent chemical stability, lower viscosity, and excellent film-forming ability, so it is suitable for application in the field of cosmetics [2,3,4,5]. In a conference of the International Federation of Cosmetic Chemists Societies in Barcelona, 1986, PFPE was first accepted as an added ingredient in cosmetics. Its function is that it can form a thin protective film to prevent corrosion from all kinds of injury [6]. Furthermore, PFPE has excellent chemical stability and corrosion resistance [7,8], so adding PFPE to cosmetics can enhance protection for outdoor workers. Moreover, PFPE has a radiation-proof effect. [9] Under the same dose, the viscosity of PFPE increases very little compared with hydrocarbon and silicon oil after the same radiation. However, because it is oleophobic and hydrophobic, it is very difficult to add PFPE to cosmetics. For PFPE delivery in cosmetics, emulsions have significant advantages in terms of cost and safety [10].

Emulsion is a multi-phase dispersive system to make one or more liquid disperse in another undissolved liquid in the form of droplets, where it is quite stable [11]. Because PFPE usually appears as an emulsion, they are named emulsion or lactescence [12]. The interfacial area of the two liquid phase increases and interfacial energy increases in the new system, so the system is thermodynamically unstable. But the mixing of the emulsifier will decrease the interfacial energy of the system, so the system will become much more stable [13]. In the emulsion, the dispersed phase is named disperse phase and the unbroken expanse is named dispersant, so the emulsion usually contains three parts: dispersant, disperse phase, and emulsifier [14]. Emulsions have a widespread use in personal care product and cosmetics to delivery nutrients to the skin and hair [15]. The stability of the emulsion [16] is an important factor affecting its application in cosmetics.

In the past years, there were researches studying the PFPE emulsions. But stability is too low to meet the requirements of practical applications. Glycerol is a commonly-used additive in cosmetics and glycerin moisturizes the skin. We chose to emulsify PFPE into glycerol [10]. We have conducted a comprehensive study of the stability of emulsions. The emulsion’s centrifugal stability, cold and heat storage stability, and its particle size change over time were studied. The rheological properties of the emulsions were tested. Compared to previous studies, the emulsions we prepared have higher stability. Finally, its application performance in detergent was evaluated [17].

## 2. Experimental Section

### 2.1. Materials

Perfluoropolyether oil (PFPE, kinetic viscosity 40 cSt) was purchased from Shanghai Aiken chemical industry Co, Ltd. (Shanghai, China). Glycerol was purchased from Tianjin No.2 Chemical Reagent Factory (Tianjin, China). Sodium dodecyl sulfate (SDS) was purchased from Changzhi Fatty Acid Factory (Changzhi, China). Sodium laureth sulfate (AES) and alkyl polyglycoside (APG) were supplied by China Research Institute of Daily Chemical Industry (Taiyuan, China). Structures of the main Material are listed in Table 1.

### 2.2. Methods and Techniques

#### 2.2.1. Preparation of Emulsions

All emulsions were prepared by the high-shear stirring emulsification method at room temperature (25 °C). Surfactant was used to stabilize the emulsion. In this study, three classic surfactants were used to obtain the emulsion. First, quantitative glycerol was weighed in a beaker. Then the surfactant was added to the beaker and stirred for 5 min. Last, PFPE was added to the beaker at high speed using a high speed homogenizer (model B25, Berthe Mechanical and Electrical Equipment Co., Ltd. Shanghai, China) at 10,000 rpm for 5 min at room temperature. The stability and droplet size were studied. All the emulsions were kept at room temperature for further study [18].

#### 2.2.2. Stability of Emulsions

This part contains centrifugal stability and storage stability. The emulsions were added to the centrifuge tube respectively; then we centrifuged the samples at 6000 r/min for 60 min. At the same time, three sealed weighing bottles filled with the three emulsions were placed into a drying beaker at 40 °C and three sealed tubes filled with the samples were placed into a refrigerator at −10–−15 °C. Last, three samples were saved at the room temperature to observe their stability for one month or more [19].

#### 2.2.3. Droplet Size

The droplet sizes of all emulsions were obtained by dynamic laser light scattering (Mastersize 2000, malvern, Worcestershire, United Kingdom) [20,21]. The emulsions were added to a beaker filled with 500 mL of deionized water until they reached the area of the detection (2 mL emulsion, approximately). we made a graph of the percentage over the distribution of particle size. The particle size was detected once per week.

#### 2.2.4. Transmission Electron Microscopy (TEM)

The distribution of particle size and micro structure of the emulsions were observed by the JEM-1011 transmission electron microscope (Joel Co., Osaka, Japan). The samples were diluted 250 times with deionized water. A drop of diluted sample was dropped onto a copper grid. Then the sample was left standing still for 10 min. Next we stained the grid, removed the excess liquid, and dried it overnight at the room temperature. Last, the grid was installed in the TEM operating to observe the sample.

#### 2.2.5. Rheological Property

The instrument is HAAKE - RheoWin (RS75, Thermo Scientific, Massachusetts, USA). We initiated the computer, started the main power, and preheated it for at least 30 min. First setting it to zero, we lifted the plate, taking bits of emulsion onto the plate that had been standing overnight to remove any bubbles. After that we made a graph of the oscillation stress sweep which revealed that the sample will not generate deformation. We used this figure to do an oscillation frequency sweep. The rheological properties can be analyzed from the graph.

#### 2.2.6. Application Performance

The prepared emulsions were added to a shampoo formulation [17]. Shampoo formulation is as follows: trisodium citrate (0.5%), dodecyl betaine (10%), APG (4%), AES (7%), glycerol (1%), PFPE emulsions (0.5%), and deionized water (additional to 100%). Among them, four kinds of shampoos were prepared—blank control (no emulsions), AES-PFPE (AES-PFPE emulsion, 0.5%), APG-PFPE (APG-PFPE emulsion, 0.5%), and SDS–PFPE (SDS–PFPE emulsion, 0.5%).Then the performance of the shampoo was tested.

(1) The Slip and Lubricity of the Emulsions

The slip and lubricity of emulsions is evaluated by the friction experiment. The friction coefficient of the cotton was measured by an Y151 type fiber friction coefficient measuring instrument to express the smoothness of the cotton. The instrument consists of a torsion balance and an electronic speed control box. The torque balance can measure the friction between the cotton and the roller mandrel (which is connected to the electronic speed control box and can be adjusted by the speed control box). First we measured the friction coefficient of the wire, then immersed it in the tested solution for 4 h, took it out and dried it at 40 ℃ for 1 h, and finally measured the friction coefficient after treatment. We queried the coefficient of friction and converted it to the coefficient of friction. 

(2) Determination of Detergency

A whiteness meter and decontamination machine (WSD-3C) was used to test the detergency. Three kinds of soiled clothes with carbon black, protein, and sebum were used in the test. The whiteness value of the stained clothes was measured before cleaning them with four kinds of detergents prepared. After drying, the whiteness value was measured again and compared with the previous value to get a comparison of detergency performances of four detergents. In this part the four detergents were dissolved in 250 ppm hard water (1 g/L).

(3) Foam Performance

Foam performance was measured by a foam instrument by the way of a modified Ross–Miles method. The four detergents were dissolved in 250 ppm hard water (1 g/L) and stored in 50 °C. Then the volume of the foam in 30 s, 3 m, and 5 m was recorded.

## 3. Results and Discussion

The emulsion was prepared by high-shear stirring at 10,000 r/min. Six grams of perfluoropolyether was dissolved in 24 g of glycerol in the presence of 0.6 g SDS (2% of the total mass). Then they were emulsified for 5 min. The other two emulsions with different surfactants were also prepared by the same way.

### 3.1. Stability of the Emulsions with Different Surfactants

The stability of the emulsion is an important factor in evaluating the emulsion. The following is an experiment to determine the emulsions’ stability.

**Figure 1:**
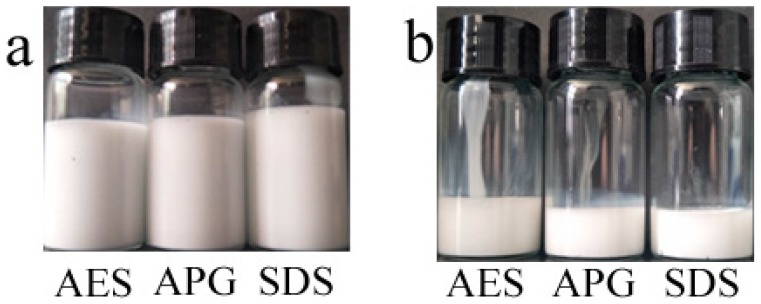

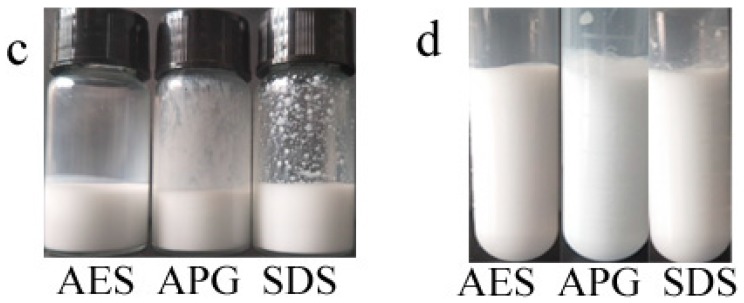
Stability of the emulsions, **a**: stored at room temperature; **b**: stored at 40 °C for 24 h; **c**: stored at −10–−15 °C; **d**: centrifugal stability.

Three surfactants (AES, APG, and SDS) were used to emulsify the PFPE. They had excellent centrifugal properties. In Figure 1d, when they were centrifuged at 6000 r/min for 60 min, they were still stable. But when the centrifugal speed was 7000 r/min, the SDS emulsion was stable; the AES and APG emulsions generated a bit of sediment. Compared with the standard [19] (centrifuged at 4000 r/min) their centrifugal property improved a lot.

Then they were subjected to an aging test. Referring to national standards (GB/T 11543-2008), firstly, three sealed weighing bottles filled with three emulsions were placed into a drying beaker at 40 °C for 24 h. Secondly, three sealed tubes filled with the samples were placed into a refrigerator at −10–−15 °C for 24 h. Last, three samples were saved at room temperature for 2 months. The results are shown separately in Figure 1a–c. Through the above three experiments, these samples were still stable—ivory liquid. It proved that the emulsion conformed to the standard of the storage stability.

The stability of the emulsion was measured macroscopically. Next we will observe the stability of the emulsion from a microscopic point of view.

### 3.2. Mean Droplet Size and Stability of Emulsion

**Figure 2:**
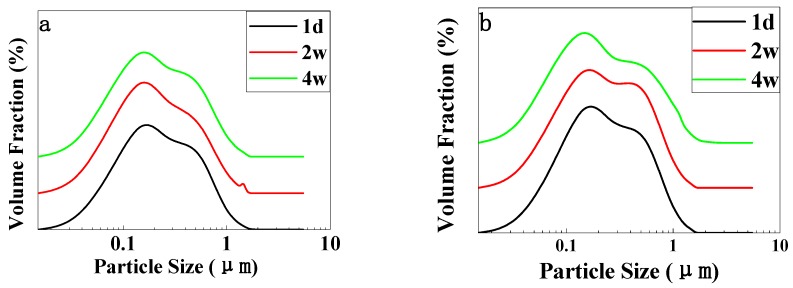

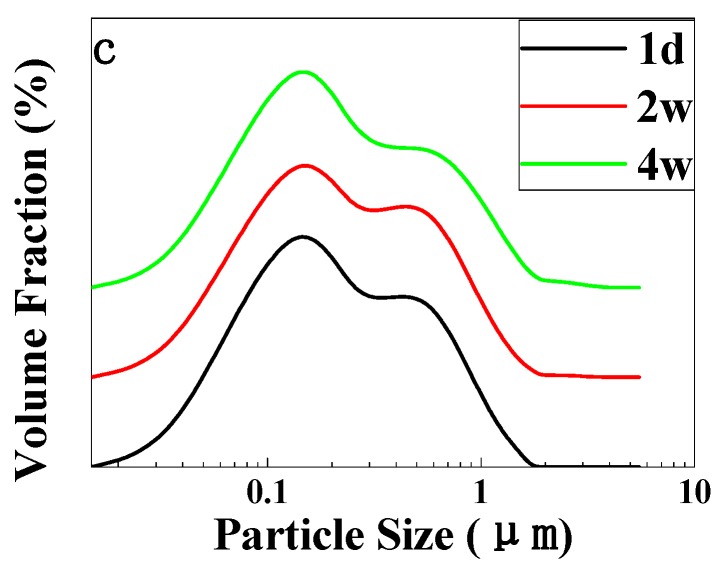
The distribution of different emulsion’s particle size over time: **a**: AES, **b**: APG, **c**: SDS.

Figure 2 depicts different emulsions’ particle size over time. The change of emulsion particle size can reflect the stability of the emulsion. As we can see from Figure 2a–c, the particle size of three emulsions was 0.03–1 μm, which indicates the emulsion’s uniformity is good. As time went on, the peak width became wider, which means the droplet size was getting bigger. However, the three figures have undergone inconspicuous changes, indicating that the particle size was stable and the stability of the emulsions was good. Moreover, in Figure 2a, the law of change is just the opposite, indicating that the droplet size of AES is smaller after one month. The mechanism still needs further study.

**Figure 3:**
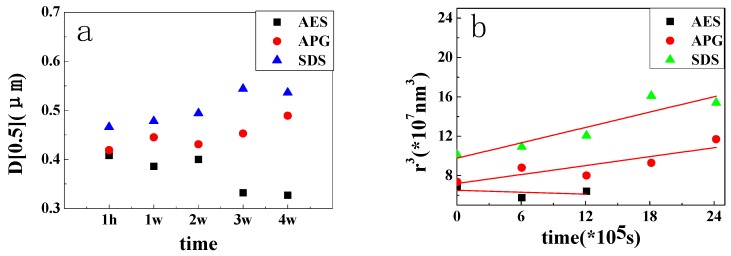
(**a**) the comparison of the mean droplet size of three emulsions over time; (**b**) the fitting curve of the mean droplet size over time.

In Figure 3, the mean droplet size of APG and SDS emulsions continued to grow. It proved that the droplet size tends to getting bigger. The oil/water interface provisionally created by the high energy input may not be fully covered with surfactant molecules, so small droplets tend to dissolve into larger ones to reduce the oil/water interface area over time. However, once the systems reach equilibrium and oil droplets are tightly encircled by surfactant molecules, the increase in droplet size tends to slow. This process could also be explained by the classical disperse system instability theory: Ostwald ripening. According to this theory, smaller drops have higher chemical potential, and larger droplets will grow at the cost of the smaller ones to reduce the potential energy of the whole system [22,23,24,25,26]. Asma Chebil et al. have reported similar results [26]. The droplet growth rate (ω) can be calculated using the following equation [25,26]:

(1) $$\omega =\frac{dr_{c}^{3}}{dt}=\frac{8DC_{\infty }V_{m}^{2}\gamma }{9RT}$$

where ω is the ripening rate, C∞ is the solubility of the dispersed phase at the planar interface, *r_c_* is the number average radius of the particles at a given time, *V_m_* is the molar volume of the dispersed phase material, *D* is the translational diffusion coefficient of the dissolved dispersed phase in the continuous phase, *γ* is the interfacial tension between the dispersed and the continuous phases, *ρ* is the density of the oil (kg/m^3^), R is the universal gas constant, and T is the absolute temperature.

Mean droplet size is the most representative point. Figure 2 shows the mean droplet size and its fitting curve. Some specific data of the fitting curve is listed in Table 2.

Figure 3b indicated that, there were good correlations between r^3^ and time, proving that Ostwald ripening is the main reason that causes the instability. The Ostwald ripening rate (ω) was obtained from the slope of every fitting curve in Table 2 [26]. From the ω, the APG emulsion is better than the SDS emulsion. Smaller ω represents more stable emulsions. The change of the AES still need further study.

### 3.3. TEM of the Emulsions

**Figure 4:**
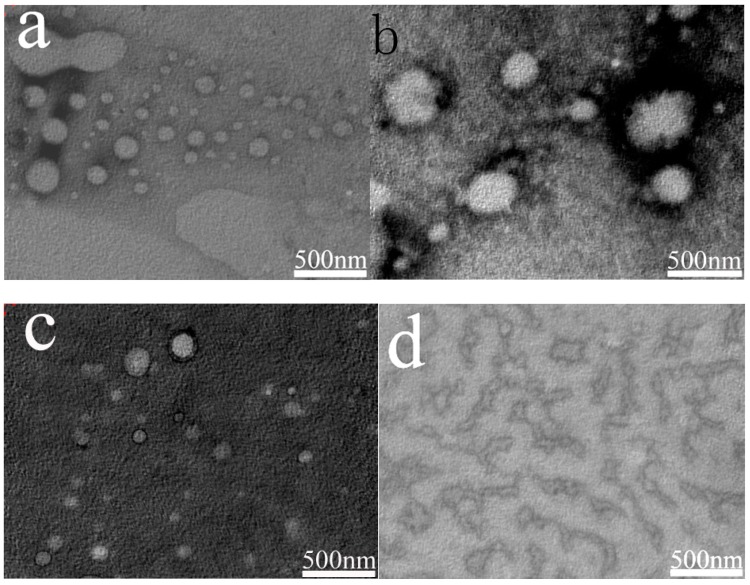

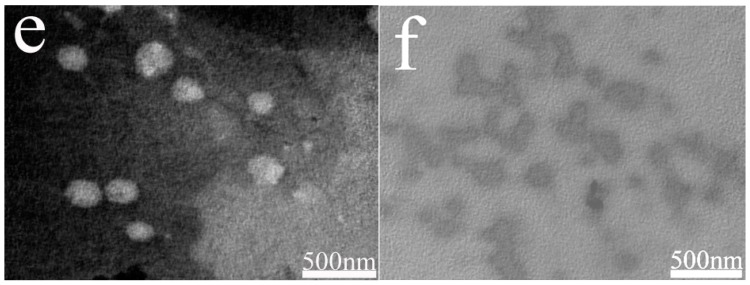
**a**, **c**, and **e** are the AES, APG, and SDS emulsions’ TEM images after one hour. **b**, **d**, and **f** are the AES, APG, and SDS emulsions’ TEM images after 3 weeks.

Figure 4 is TEM of three emulsions. From the TEM image, we can more intuitively feel the microstructure and change of particle size of the emulsion droplets.

As time passed, the microstructure of APG and SDS emulsions changed. As we can see in the Figure 4, small droplets began to gather because of the effect of Ostwald ripening. In the SDS emulsion (Figure 4f), the particle size is bigger than it in the APG emulsion (Figure 4d). But in Figure 4a,b, the particle size changed a little. In the figure, there are still many emulsion particles with small particle size, and there is no large-scale particle polymerization phenomenon. It proved that the order of the stability is AES > APG > SDS.

### 3.4. Rheological Properties

**Figure 5:**
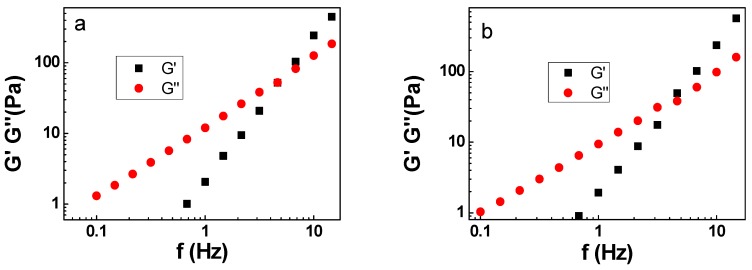

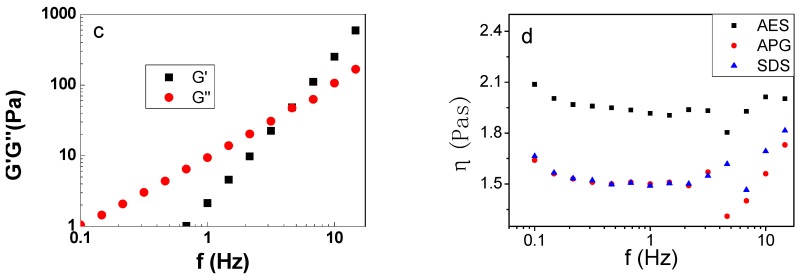
The viscoelasticity and viscosity as a function of shear rate of three emulsions **a**: AES, **b**: APG, **c**: SDS, **d**: the viscosity of three emulsions.

The rheological properties of the emulsion will affect its performance in applications. Figure 5 shows the viscoelasticity and viscosity as a function of shear rate of three emulsions.

In AES, as viscosity decreased with increasing shear frequency, it proved that the AES emulsion is pseudoplastic colloids. The viscosity of the pseudoplastic colloids decreases with the increase of the shear rate because of the presence of flocculation and demagnetization in the dispersion. Under the action of shear, the flocculation is damaged, making the system into a mobile state. Therefore, increasing the shear rate and destroying the colloidal structure can result in a decrease in viscosity. In APG and SDS emulsions, the viscosity had no significant changes over frequency, proving that its viscosity remained stable. In addition, the viscosity of AES emulsion is larger than the viscosity of the others. According to the Stokes formula, the higher the viscosity, the slower the phase separation. The viscosity of emulsion is related to its stability. So the AES emulsion is more stable than the others.

In Figure 5a–c, G’ and G’’ had an intersection. In 0.01–5 Hz, the G’’ of the emulsions are all greater than G’, showing that the liquid-like viscosity is dominant. In 5–12 Hz, the G’ of the emulsions are all greater than G’’, showing elasticity predominating. It shows that the emulsion we prepared was a viscoelastic system based on stickiness and emulsion fluidity was good.

### 3.5. Application in Shampoo of the Emulsions

In practical application, many factors of a product will affect consumers’ purchasing choices, for example, safety parameters, sensorial parameters, foam quality parameters, and cleaning parameters. The basic function of shampoo is cleaning. Due to the limitation of experimental conditions, this paper studied the application performance of the product from the following three aspects.

#### 3.5.1. The Slip and Lubricity of the Emulsions

The slip and lubricity of emulsions was evaluated by the friction experiment. The improvement of fabric softness by emulsion was measured by the friction coefficient. The cotton’s friction coefficient was measured before and after treatment to get a conclusion that whether the emulsions affect the slip and lubricity of the cotton. Friction coefficients of cotton after processing by emulsions were listed in Table 3.

In this Table 3, the AES-PFPE emulsion’s effect on the smoothness of the cotton is the most obvious. The difference between before and after is as high as 45%, meaning that the cotton’s friction coefficient was improved a lot after treatment by AES-PFPE emulsion.

In Table 4, the cotton’s friction coefficient was measured after treatment by water and a diluted AES–PFPE emulsion. It proved that the water has no effect on the change of the friction coefficient. The diluted emulsion can reduce the cotton’s friction coefficient and improve the slip and lubricity of the cotton.

#### 3.5.2. Effect of the Emulsions on the Detergency of Different Shampoo

Perfluoropolyether (PFPE) is another kind of special liquid material. It is colorless, tasteless, no-poisonous, hypo-allergenic, and has excellent handling properties, excellent chemical stability, lower viscosity, and excellent film-forming ability, so it is suitable for application in the field of cosmetics.

Because of the emulsions’ good dispersion in water, emulsions were added to the shampoo to test its application performance. The shampoo formulation was as follows: trisodium citrate (0.5%), dodecyl betaine (10%), APG (4%), AES (7%), glycerol (1%), and deionized water (additional to 100%). Among them, four kinds of detergent were prepared-–blank control (no emulsions), AES-PFEP (AES-PFPE emulsion, 0.5%), APG-PFPE (APG-PFPE emulsion, 0.5%), and SDS-PFPE (SDS-PFPE emulsion, 0.5%).

Decontamination ability is an important symbol to evaluate the quality of shampoo. The most basic function of shampoo is cleaning. It can remove excess grease and dirt from the scalp.

(2) $$\mathrm{Decontamination}\;\mathrm{Index}=\frac{\mathrm{whiteness}\;\mathrm{of}\;\mathrm{sample}}{\mathrm{whiteness}\;\mathrm{of}\;\mathrm{standard}}$$

In Table 5, the decontamination index was close to 1.00, indicating the addition of the emulsions did not affect the detergency of the shampoo. Among them, the addition of the AES-PFPE emulsion had a promoting effect in removing protein. In general, the addition of three emulsions did not affect the detergency performance of the shampoo itself. In addition, three emulsions had no obvious difference in detergency performance.

#### 3.5.3. Effect of the Emulsions on the Foam Performance of Different Shampoo

The foam ability of shampoo is the most sensitive choice indicator for consumers, so we explored whether the addition of the emulsion will affect the foaming performance of shampoo. In Table 6, the foam volume of the three samples was comparable to the blank sample, indicating that the addition of three emulsions did not affect the foam performance of the shampoo. Moreover, the foam performance and stability was better when we added the SDS-PFPE emulsion to the shampoo.

## 4. Conclusions

In this paper, perfluoropolyether oil was successfully emulsified and added into a shampoo formulation, and its performance was tested. The emulsions prepared with the three surfactants we chose have respective advantages. In the centrifugal property, the SDS emulsion is the best, and the storage stability of the three emulsions has reached the national standard.

We drew a conclusion that the main mechanism for instability was Ostwald ripening by analyzing the mean droplet size over time. The Ostwald ripening rates of the three emulsions were different. AES is the slowest, followed by APG, and finally SDS. Through analysis of their rheological properties, three kinds of emulsions can be said to be shear thinning fluids as they meet the requirements for application. Compared to previous work, these emulsions are more stable.

The diluted AES-PFPE emulsion can improve the slip and lubricity performance of cotton. In the actual formulation system, the addition of three emulsions will not affect the shampoo’s detergency and foam performance. In certain aspects, the improved detergent has a better performance. For example, SDS-PFPE emulsion has better centrifugal stability and the detergent with added SDS-emulsion has better foaming performance and foam stability. The AES emulsion is the most stable emulsion and its detergency is the best among them. In general, AES emulsions have the best performance in three emulsions.

## Figures and Tables

**Table 1:** Structure of the main Material. PFPE: perfluoropolyether; AES: sodium laureth sulfate; APG: alkyl polyglycoside; SDS: sodium dodecyl sulfate.

Material	Structure
PFPE	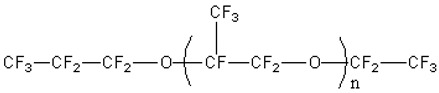
AES	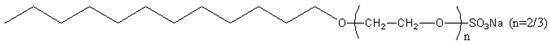
APG	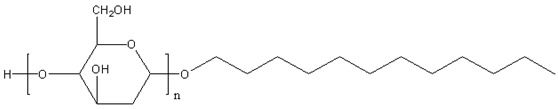
SDS	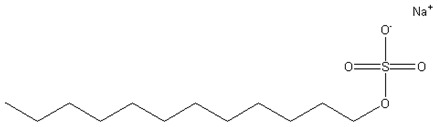

**Table 2:** Ostwald Ripening Rate (ω) of the Emulsions.

	AES	APG	SDS
ω (*10^2^nm^3^/s)	−6.51032	7.19765	9.76552
R	0.72454	0.67792	0.81203

**Table 3:** Friction coefficient of cotton after processing by emulsions.

		Friction Coefficient					
		1	2	3	4	5	Average
AES-PFPE	Before treatment	0.6886	0.9233	0.5835	0.7208	1.0672	
	After processing	0.5046	0.7331	0.3833	0.5205	0.5835	
	Rate of decline (%)	26.72	20.6	34.31	25.94	45.32	30.58
APG-PFPE	Before treatment	0.6751	0.5373	0.5737	0.555	0.5288	
	After processing	0.604	0.5046	0.4894	0.5288	0.6621	
	Rate of decline (%)	10.53	6.09	14.69	4.72	−25.21	9.01
SDS-PFPE	Before treatment	0.626	0.5288	0.5205	0.6148	0.626	
	After processing	0.5205	0.5288	0.3388	0.4351	0.4969	
	Rate of decline (%)	16.9	0	34.91	29.23	20.62	25.42

**Table 4:** Friction coefficient of cotton after processing by diluted AES-PFPE emulsion.

		Friction Coefficient					
		1	2	3	4	5	Average
water	Before treatment	0.1818	0.1574	0.2021	0.1627	0.2144	
	After processing	0.1875	0.1627	0.2113	0.1627	0.2113	
	Rate of decline (%)	0	0	0	0	0	0
10%	Before treatment	0.1904	0.1734	0.1707	0.1904	0.2021	
	After processing	0.1734	0.1574	0.1548	0.1762	0.1680	
	Rate of decline (%)	8.93	9.23	9.31	7.46	16.87	10.36
30%	Before treatment	0.2021	0.1707	0.1992	0.1653	0.2144	
	After processing	0.1627	0.1653	0.1818	0.1734	0.1600	
	Rate of decline (%)	19.5	3.16	8.73	0	25.37	14.19
60%	Before treatment	0.1790	0.1846	0.1707	0.1962	0.1600	
	After processing	0.1790	0.1653	0.1992	0.1790	0.1421	
	Rate of decline (%)	0	10.46	0	8.77	11.19	10.14

**Table 5:** Decontamination index of various emulsions.

	C_1_	P_1_	S_1_	C_2_	P_2_	S_2_	Average		
							C	P	S
AES-PFPE	0.966	0.963	1.110	0.992	1.090	0.891	0.979	1.0265	1.0005
APG-PFPE	0.995	1.019	0.956	1.006	0.973	0.931	1.0005	0.996	0.9435
SDS-PFPE	0.971	0.977	1.016	1.037	1.027	0.920	1.0004	1.002	0.968

(C:carbon, P:protein, S:sebum).

**Table 6:** Foam property test of emulsions with various surfactants.

Volume (mL)	Blank Control		AES-PFPE		APG-PFPE		SDS-PFPE	
	1^#^	2^#^	1^#^	2^#^	1^#^	2^#^	1^#^	2^#^
30s	340	330	320	320	330	310	330	340
3m	325	310	320	308	316	310	320	330
5m	315	310	310	298	315	305	320	330
